# A new questionnaire to determine the effect of team interaction in the ICU on perceived futility and intention to quit: results of a pilot study in two German hospitals

**DOI:** 10.1186/cc13221

**Published:** 2014-03-17

**Authors:** D Schwarzkopf, F Bloos, A Meier-Hellmann, C Icke, N Riedemann, CS Hartog

**Affiliations:** 1 Jena University Hospital, Jena, Germany; 2 Helios Hospital Erfurt, Germany

## Introduction

Perceived futility of care may jeopardize patient quality of care and increase ICU staff turnover. It is related to workload and interdisciplinary collaboration. Our aim was to evaluate concepts from team science, namely inclusive leadership (a style that invites and appreciates others' contributions) and psychological safety (a key antecedent of speaking up and learning behavior), and to determine their effect on perceived futility and intention to quit.

## Methods

A staff survey in four interdisciplinary ICUs and two intermediate care units of two tertiary care hospitals. The questionnaire contained validated scales to assess inclusive leadership of head nurses and attending physicians, nurse-physician collaboration, collaborative decision-making, psychological safety, workload, intention to quit and a newly developed scale for perceived futile care. English questions were translated into German by state-of-the art forward and backward translations. Reliability - that is, internal consistency and inter-rater reliability of scales - was evaluated. Predictors of perceived futile care and intention to quit were identified by multiple regressions.

## Results

A total of 220 nurses and 55 physicians participated. Scales showed good reliability with all Cronbach's alpha ≥0.8 and significant between unit variability (all *P *≤ 0.011). On a scale from 0 (‘never') to 5 (‘very often'), ICU staff rated frequency of futile care as median 3.6 (IQR: 3 to 4). On a seven-point Likert scale with 7 denoting maximal values, intention to quit was rated as 2.3 (1 to 4). Nurses gave significantly lower ratings of job satisfaction than physicians (4 (3 to 5) vs. 5 (4 to 6), *P *≤ 0.001), perceived futility more often than physicians (*P *≤ 0.001), and rated all aspects of nurse-physician collaboration worse than physicians, including attendings' inclusive leadership, collaboration, decision-making and psychological safety (all *P *≤ 0.001). Futility and intention to quit were each predicted by high workload and low psychological safety (all *P *≤ 0.009). Among nurses, inclusive leadership by the head nurse prevented intention to quit (*P *= 0.001) and a composite score of good nurse-physician interactions predicted less perception of futile care (*P *≤ 0.001).

## Conclusion

The scales of the new questionnaire showed good reliability and differentiated between nurses and physicians. Psychological safety and inclusive leadership proved to be important concepts.

